# Empowering primary care physicians in child and adolescent psychiatry: a needs assessment on collaborative care in Dubai

**DOI:** 10.3389/fmed.2024.1456212

**Published:** 2024-11-11

**Authors:** Anna Berbenyuk, Asma Alameeri, Asma Bin Ismail, Nabil Zary, Meshal A. Sultan

**Affiliations:** ^1^Institute of Learning, Mohammed Bin Rashid University of Medicine and Health Sciences, Dubai Health, Dubai, United Arab Emirates; ^2^Primary Health Care, Dubai Health, Dubai, United Arab Emirates; ^3^College of Medicine, Mohammed Bin Rashid University of Medicine and Health Sciences, Dubai Health, Dubai, United Arab Emirates

**Keywords:** primary care physicians, child and adolescent psychiatry, collaborative care, educational program, Dubai

## Abstract

**Background:**

Child and adolescent psychiatric disorders pose significant public health concerns necessitating prompt intervention. Primary care physicians (PCPs) play a critical role as initial points of contact, facilitating early detection, management, and referral of these conditions. In Dubai, integrating mental health services into primary care faces unique challenges, highlighting the need for systemic reforms and enhanced PCP training.

**Objective:**

This study investigated perceptions, barriers, and systemic challenges encountered by PCPs in managing child and adolescent psychiatric conditions in Dubai’s primary care setting. It also assessed family physicians’ involvement and preparedness in this domain.

**Methods:**

Using a mixed-methods approach, we conducted a survey among family physicians in Dubai Health facilities and analyzed patient data from family medicine clinics. The survey evaluated formal training, preparedness, encounter frequency, and referral patterns for psychiatric care in young patients. Qualitative insights from open-ended survey questions provided additional understanding of specific challenges.

**Results:**

Findings revealed significant gaps in formal training, with only 33.3% of respondents trained in child and adolescent psychiatry during medical education, and 21.1% participating in Continuing Medical Education (CME). A majority (54.4%) of PCPs felt unprepared to manage psychiatric care in young patients. Patient data analysis showed a predominance of male patients (59.9%) and identified Autism Spectrum Disorder (43.1%) as the most common condition, emphasizing the reliance on specialized care through a high referral rate (63.1%). Major barriers included time constraints, limited psychiatric knowledge, resistance to mental health services, systemic and structural issues, communication challenges, and resource shortages.

**Conclusion:**

Enhanced training and systemic reforms are urgently needed to integrate mental health services effectively into Dubai’s primary care. Implementing structured collaborative care models, fostering interdisciplinary collaboration, and addressing systemic barriers are crucial for improving child and adolescent psychiatric condition management. These initiatives promise better patient outcomes and more efficient healthcare resource utilization.

## 1 Introduction

The mental health of children and adolescents is a major healthcare focus due to its significant influence on their growth and well-being. Among the various mental health conditions, Attention-Deficit/Hyperactivity Disorder (ADHD) and Autism Spectrum Disorder (ASD) are increasingly common. ADHD is one of the three most common disorders encountered in primary care settings (1). While the detection of ASD has increased threefold over the past two decades (2). Primary care providers (PCPs) play a central role in diagnosing and managing psychiatric disorders, yet many face challenges due to insufficient knowledge, skills, or time. This gap can lead to long-term negative consequences for patients, highlighting the need for effective interventions to support PCPs in their role (3).

Despite effective treatments, fewer than half of youth with diagnosed mental health disorders receive care due to a shortage of specialists, particularly child and adolescent psychiatrists, in the United States (4). Over half of pediatric primary care visits address mental health issues, with primary care clinicians prescribing most psychotropic medications for youth (5, 6). Commonly encountered disorders include anxiety, depression, and ADHD, most of which are mild to moderate (5, 7, 8). Collaborative partnerships between primary care clinicians and mental health specialists can help manage these issues by providing necessary support in a personalized, long-term, and family-centered manner. These partnerships can significantly expand the mental health workforce and help bridge the gap between the millions of youths needing effective mental health services and those receiving them (5).

In many countries, including the United Arab Emirates (UAE), primary care serves as the initial point of contact for individuals with mental health challenges. It is within these settings that the majority of mental health problems are addressed, integrating physical and emotional care in a less stigmatizing environment (9). However, despite their frontline role, many PCPs are not adequately trained or supported to recognize and treat mental health conditions effectively, often resulting in untreated or poorly managed cases until they require more urgent psychiatric care (9).

The Global Mental Health (GMH) Movement emphasizes the critical need to improve access to mental health care and reduce treatment gaps, particularly for children and youth (10). In the UAE, this challenge is evident as PCPs are often the primary providers of mental health services due to a scarcity of specialized mental health professionals. Consequently, there is a pressing need to enhance the capabilities of PCPs through education and collaborative care models.

Collaborative care involves the integration of primary care and mental health services, where providers share resources, expertise, and decision-making responsibilities to deliver person-centered, effective, and timely care (9). This approach has shown promise in addressing the barriers faced by PCPs, enabling them to manage mental health conditions more effectively within primary care settings. Collaborative care models, though not yet widely implemented in Dubai, serve as an important framework for improving mental health care in the region. This approach, inspired by Canadian healthcare practices, has seen more structured implementation in Abu Dhabi, where mental health services are integrated into primary healthcare centers (PHCs). While Dubai is still in the early stages of adopting such models, the potential for scaling is significant.

Social determinants of health, such as poverty and associated factors like parental illness and transient housing, further complicate the diagnosis and management of mental illnesses among children (11). Innovative collaborative practice models that integrate child psychiatry consultations with community-based family support specialists (FSS) have demonstrated higher rates of access and engagement, suggesting that a family-centered, integrated approach can mitigate these barriers and improve outcomes for at-risk youths (11).

Despite the likely effectiveness of collaborative care, there remains a significant gap in its implementation and outcomes, particularly concerning ADHD management. Current studies do not provide strong evidence for the effectiveness of collaboration between PCPs and mental health professionals in this context, underscoring the need for further research and innovation (3). Future studies should focus on educating PCPs about management guidelines and exploring novel methods of collaboration that directly enhance patient care.

In the UAE, empowering PCPs through collaborative care and targeted education is essential to bridge the gap between primary healthcare and specialized psychiatric services. This research project aims to assess and enhance the capabilities of PCPs in diagnosing, managing, and providing care for child and adolescent psychiatric cases. By conducting a comprehensive needs assessment survey and analyzing patient records, the study seeks to develop effective interventions that improve mental health services within primary care settings, ultimately benefiting the young population in the UAE.

### 1.1 Aim

This research’s aim was to assess the educational needs of family physicians in child and adolescent psychiatry, considering the specific challenges and complexities of handling these cases in primary care. Additionally, the study aimed to evaluate existing referral patterns and trends in cases managed within primary care settings.

### 1.2 Objectives

(1) To conduct a needs assessment survey at primary healthcare clinics (PHCs) under Dubai Health, with the goal of assessing the prior training and current expertise of general practitioners in child and adolescent psychiatry, as well as identifying knowledge gaps and challenges.
(2) To conduct an in-depth analysis of data related to child and adolescent psychiatry care in the primary care setting, by retrieving relevant information from the medical information system, including data on disease prevalence, follow-up records, and referral patterns.

## 2 Materials and methods

### 2.1 Study design, Setting, and participants

This study was conducted within the PHCs of Dubai Health, an integrated academic healthcare system. Study methodology employed a mixed-methods design, incorporating both quantitative and qualitative approaches to comprehensively assess the needs and perspectives of family physicians regarding child and adolescent psychiatric care within primary care settings in Dubai. Additionally, patient data was analyzed to gather objective insights into most frequently encountered diagnoses and referral patterns. Family physicians were defined as physicians who have completed a medical residency program in family medicine. Primary care facilities managed by Dubai Health serve as the initial point of contact for medical care in Dubai, UAE, for both adult and pediatric populations.

The study spanned a duration of 5 months, with data collection running from January 29, 2024 to April 30, 2024, and final data analysis completed in June 2024. Adherence to the STROBE guidelines was maintained in the reporting of this study (12).

Participants in this study included all family physicians currently employed within the Dubai Health network. No exclusion criteria were applied during the recruitment process for family physicians. Additionally, data from electronic medical records on children and adolescents aged between 6 and 18 years diagnosed with psychiatric conditions between November 1, 2023 and April 30, 2024 was collected.

### 2.2 VariablesThe study examined the following variables:

(1) Demographic and professional characteristics of family physicians: Age, gender, years of experience, qualifications, and training in child and adolescent psychiatry.
(2) Knowledge and practices related to child and adolescent psychiatry: Self-reported knowledge gaps, referral practices, and perceived barriers to providing psychiatric care.
(3) Qualitative data: Themes and patterns from open-ended survey responses.
(4) Patient-related data: Age, gender, psychiatric diagnosis, and referral routes for children and adolescents presenting to the primary care setting.

### 2.3 Data sources/measurement

(1) Survey

Data was collected through a computer-based survey (see Supplementary Table 1) using Microsoft Forms. The survey, designed in English, was distributed via email to all family physicians within the Dubai Health network. The survey was designed by the researcher (A.B.) and validated for content relevance by other team members (M.S., N.Z., A.A.). The survey included both closed-ended questions to gather quantitative data and open-ended questions to elicit qualitative insights. The survey was estimated to take approximately 11 min to complete.

(2) Patient Records Data Extraction

Patient data was extracted from the electronic medical records system for all PHCs under Dubai Health. Data points included patients’ age, gender, psychiatric diagnosis, and referral routes. The extracted data were anonymized and systematically organized in Excel spreadsheets for analysis (see Supplementary Table 2).

To obtain a comprehensive understanding of physicians’ training in child and adolescent psychiatry and referral patterns, quantitative and qualitative survey data and patient records data was triangulated.

### 2.4 Study size

**FIGURE 1:**
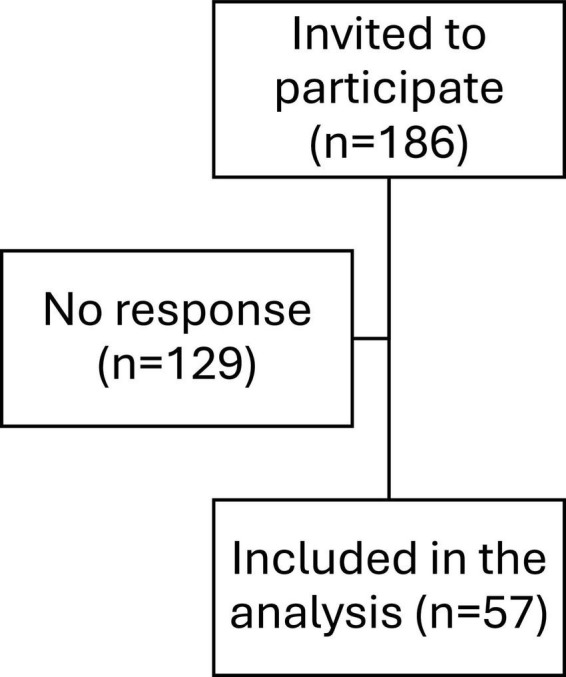
Flow chart of survey participants (*n* = 57).

All interested family physicians within Dubai Health who gave consent were eligible to participate in the study. Thus, no sample size was statistically calculated as it was based on obtaining complete coverage. The final sample size was 57 physicians (Figure 1). Furthermore, consent for using information related to their children’s clinical encounters was waived as it is included in the general consent signed upon clinic admission. Data was included from electronic medical records of all children and adolescents aged 6–18 years diagnosed with a psychiatric disorder by a family physician during the period from November 1, 2023 to April 30, 2024. The total number of included records was 382.

### 2.5 Statistical methods

Statistical analysis was performed using the Statistical Package for Social Sciences (SPSS), version 29. A significance level of *p* ≤ 0.05 was established.

Quantitative data analysis involved employing descriptive statistical techniques to summarize both survey responses and patient record information. This encompassed calculating means, standard deviations (SD), frequencies, and percentages for survey responses and patient demographics. Additionally, an in-depth analysis of referral practices and diagnostic trends was conducted to identify patterns.

In addition to descriptive analysis, inferential statistical techniques were employed to examine relationships within the data. A Chi-square test was used to assess associations between categorical variables. In this study, we developed a structured survey to assess key aspects of participants’ experiences, training, and referral behaviors in child and adolescent psychiatric care. Given the diversity of question types, including dichotomous and Likert-type items with varying scales, formal internal consistency measures such as Cronbach’s alpha were not calculated. Instead, the survey’s content validity was ensured through expert input, which guided the design to cover all relevant domains comprehensively. Adequate response rates and consistent patterns observed across related items further contribute to the survey’s reliability.

The qualitative part of the study utilized a phenomenological research design, grounded in constructivist epistemology, to guide the interpretive qualitative analysis. The analysis followed Braun and Clarke (13) six-step approach, an inductive multi-stage methodology recommended for qualitative socio-behavioral research and endorsed for health professionals’ education research (14). This participant-centered design enabled the researchers to deeply explore the participants’ lived experiences.

Qualitative data analysis began after the data collection phase concluded, employing an inductive approach grounded in constructivist epistemology. The analysis was iterative and conducted using a participant-focused, phenomenological approach to thematic analysis by two data analyzers (M.S. and A.B.). Prior to analysis, the analyzers identified personal characteristics they believed might influence their perceptions of the subject matter. Consistency in underlying assumptions and theories was maintained by an independent facilitator (F.O.), who has expertise in qualitative socio-behavioral research. This interpretative approach differs from standard scientific inquiry, focusing on understanding and relating to individuals’ thoughts, ideas, motives, aspirations, and actions, rather than finding causal explanations. It assumes that we can interpret individuals’ thoughts, emotions, and behaviors by actively listening to and understanding their self-expressions.

The adapted qualitative analysis followed Braun and Clarke’s six-step framework, a widely used methodology in health professions’ education research. NVivo software version 12.0 plus (QSR International Pty. Ltd., Chadstone, Australia) was utilized to code the data and facilitate the categorization of text fragments.

The analysis process involved the following steps:

(1) Familiarization with the data: The data analyzers worked as a group, reading the survey responses aloud to familiarize themselves with the dataset, reflecting on the content, and sharing their thoughts and ideas.
(2) Generation of initial codes: Relevant text fragments from the responses were extracted and tagged based on their relation to the study’s purpose, particularly the challenges and possible solutions identified by the participants, continuing until data saturation was achieved.
(3) Searching for themes: Several rounds of structured reflections were conducted to identify potential interconnections among the identified categories.
(4) Reviewing themes: Categories were collated into higher-order themes according to logical linkages identified by the data analyzers.
(5) Defining and naming themes: Categories and themes were coded, labeled, and defined, resulting in the study’s conceptual model.
(6) Reporting on findings: The results were reported narratively in alignment with established guidelines, such as the Standards for Reporting Qualitative Research (SRQR) (15). A tally was conducted to substantiate the findings, reflecting the number of participants mentioning each category.

## 3 Results

**FIGURE 2:**
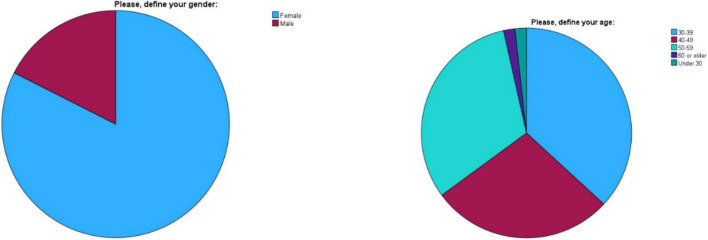
Demographic characteristics of family physicians participating in the survey (*n* = 57).

A total of 186 family physicians employed in Dubai Health were approached for the survey, with 57 responding, resulting in a response rate of 30.6% (Figure 1). Among the respondents, 47 were female (82.5%) and 10 were male (17.5%). The age distribution was as follows: 21 participants (36.8%) were aged 30–39, 16 participants (28.1%) were aged 40–49, 18 participants (31.6%) were aged 50–59, one participant (1.8%) was 60 or older, and one participant (1.8%) was under 30 (Figure 2). The years of practice as family physicians ranged from 0 to 33 years, with a mean of 13.84 years (SD = 7.93). Participants worked in various clinics, with the highest representation from Nad Al Hamar (28.1%), followed by Al Barsha (17.5%) and Al Mizhar (14.0%), which are the biggest within the network.

### 3.1 Family physician’s survey

Given the diverse structure of the questions, including dichotomous and Likert-type items with varying response scales, traditional measures of internal consistency such as Cronbach’s alpha were not calculated. Similarly, exploratory factor analysis was deemed inappropriate due to the heterogeneity of the question formats. Despite this, the survey design was carefully reviewed to ensure content validity, with expert input guiding the selection of items to effectively capture the relevant domains of training and preparedness.

The structured approach and adequate response rates suggest that while the survey presents opportunities for improvement in internal consistency, it demonstrates a reasonable degree of reliability and construct validity.

Regarding training in child and adolescent psychiatry, 19 respondents (33.3%) reported receiving training during medical school, residency, or fellowship, while 38 respondents (66.7%) did not. For continuing medical education (CME) in child and adolescent psychiatry, 12 respondents (21.1%) reported receiving training. When asked about their involvement in collaborative sessions between primary and secondary care physicians, only 14 respondents (24.6%) reported having participated. When asked about their preparedness to manage child and adolescent psychiatric care, 4 respondents (7.0%) felt not prepared at all, 27 respondents (47.4%) felt not very prepared, 23 respondents (40.4%) felt somewhat prepared, and 3 respondents (5.3%) felt very prepared. The majority of respondents (89.5%) expressed interest in collaborative care educational initiatives or interdisciplinary teams for managing child and adolescent psychiatric care.

**FIGURE 3:**
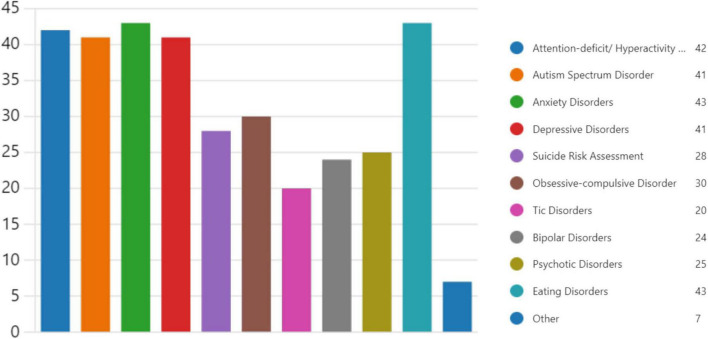
Family physicians’ reported interest in topics for further training (*n* = 57).

In terms of encountering child and adolescent patients with psychiatric concerns, 4 respondents (7.0%) very frequently (weekly), 17 respondents (29.8%) reported encountering them frequently (1–3 times per month), 19 respondents (33.3%) sometimes (around once a month), and 17 respondents (29.8%) occasionally (1–3 times per year). The typical course of action when encountering a child or adolescent patient with significant psychiatric needs varied: 6 respondents (10.5%) referred to specialized care immediately, 86% respondents (conducted a primary assessment and referred to specialized care if needed, and one respondent (1.8%) managed the case themselves. Additionally, one respondent covered the adolescent clinic in primary health care clinic. Regarding the percentage of cases referred to specialized care, 23 respondents (40.4%) reported referring over 50% of cases, 6 respondents (10.5%) referred 30–50% of cases, 17 respondents (29.8%) referred 15–30% of cases, while 10 respondents (17.5%) referred 0–15% of cases. Only one respondent (1.8%) did not provide an estimate as they reported managing the case themselves (Figure 3).

**FIGURE 4:**
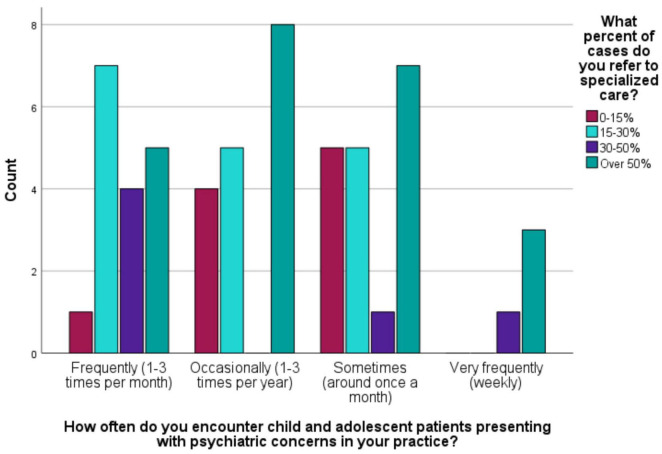
The frequency of encountering child and adolescent psychiatry cases and the percentage of cases referred to specialized care (*n* = 57).

Training during medical school, residency, or fellowship, and CME training were assessed in relation to age groups. The majority of participants in the 30–39 and 50–59 age groups did not receive training during medical school or residency. However, higher percentages in older age groups reported receiving CME training. Participation in collaborative sessions was highest among the 40–49 age group. Preparedness to manage psychiatric care varied by training background. Contrary to our initial interpretation, the majority of participants who received training during medical school or through continuing medical education (CME) still felt unprepared to manage psychiatric care. In contrast, a larger proportion of participants without such formal training reported feeling somewhat or very prepared (Table 1). This unexpected finding suggests that other factors, such as personal experiences or informal learning, may influence perceived preparedness. Family doctors were asked to identify areas they would like to receive additional training in, and the top five topics were anxiety disorders (75.4%), eating disorders (75.4%), ADHD (73.7%), autism spectrum disorder (71.9%), and depressive disorders (71.9%) (Figure 4).

**TABLE 1:** Preparedness to deal with child and adolescent psychiatry cases and prior training (*n* = 57).

Variable	Do you feel adequately prepared to manage child and adolescent psychiatric care in your practice?				Total	*p*-value for the Pearson’s Chi-square test*
	**Not prepared at all**	**Not very prepared**	**Somewhat prepared**	**Very prepared**		
**Training or education during your medical school/residency/fellowship**						
Yes	3	23	11	1	38	0.024
No	1	4	12	2	19	
Total	4	27	23	3	57	
**Training or education as part of continuing medical education (CME)**						
Yes	4	25	16	0	45	<0.001
No	0	2	7	3	12	
Total	4	27	23	3	57	

*A significance level of *p* ≤ 0.05 was established.

### 3.2 Qualitative analysis

As per the qualitative findings, family physicians’ training in child and adolescent psychiatry varied widely, spanning medical school, residency, and fellowship programs, as well as CME. During formal education, exposure included rotations and specific attachments, such as a 2-week adolescent clinic attachment or month-long rotations in pediatric psychiatry. Advanced degrees and diplomas, such as those in family medicine and applied mental health, provided further specialized training. Continuous education was maintained through online courses and attendance at psychiatric conferences and workshops.

Collaborative sessions that were reportedly attended by some participants covered a range of psychiatric conditions, including depression, anxiety, and bipolar disorder. Other topics covered in collaborative sessions included early disease detection, management, and referral practices, gastroenterology, orthopedics, and diabetes care.

**FIGURE 5:**
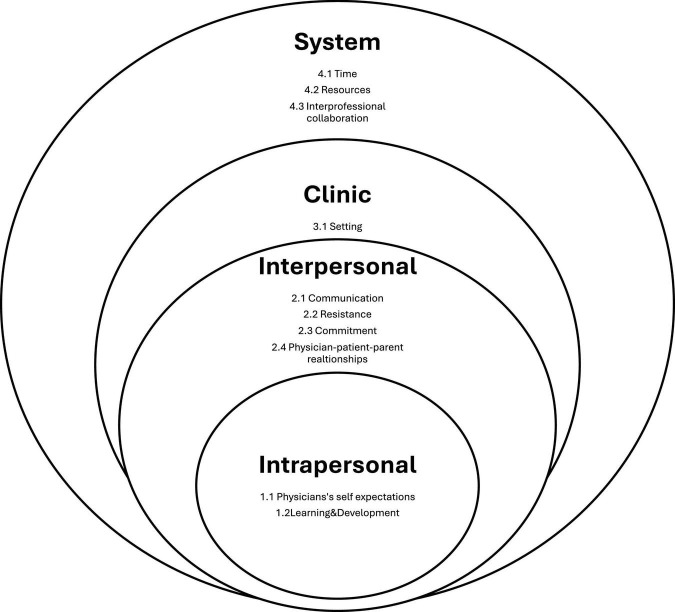
Conceptual framework of current study: “perceived family physicians’ challenges and barriers in child and adolescent psychiatry care.”

High number of responses (57) for open-ended questions around challenges and areas for improvement in child and adolescent psychiatry indicates a high importance of the topic to family physicians. The survey revealed several key themes from the responses of family physicians regarding their experiences and challenges in child and adolescent psychiatry. Each theme includes specific categories that provide a deeper understanding of the physicians’ perspectives and practices (Figure 5 and Table 2).

**TABLE 2:** Semi-quantitative tally of the output of the participant-focused qualitative analysis.

Theme	Intrapersonal		Interpersonal				Clinic	System		
**Category**	**Physicians’ self-expectations**	L&D*	**Communication**	**Resistance**	**Commitment**	**P-P-P relationship**	**Setting**	**Resources**	**Interprofessional collaboration**	**Time**
Sum	8	33	10	9	3	12	5	25	18	24

*L&D, learning and development; P-P-P, physician-patient-parent.

#### 3.2.1 Theme: intrapersonal

This theme relates to the internal aspects of physicians’ professional lives, focusing on their personal standards, growth, and self-expectations in the field of child and adolescent psychiatry.

*Physicians’ Self-Expectations*

Family physicians expressed their personal aspirations concerning their competency and level of knowledge and skills in handling child and adolescent psychiatry presentations. Many discussed the lack of knowledge and skills required to handle these cases as well as the need for specific training, like cognitive-behavioral therapy (CBT), reflecting a continuous desire for personal and professional growth and willingness to cover the gaps in care.

*Learning and Development (L&D)*

Ongoing education and training emerged as essential for physicians to stay current with the latest developments in child and adolescent psychiatry. They emphasized the importance of continuous learning opportunities to enhance their skills and knowledge, which in turn boosts their confidence and effectiveness in treating young patients.

#### 3.2.2 Theme: interpersonal

This theme encompasses the dynamics and interactions between physicians, patients, and their families, emphasizing the importance of communication and relationships.

*Communication*

Effective communication was identified as a critical component of patient care. Physicians highlighted the challenges and strategies involved in conveying complex psychiatric information in a clear, supportive manner. Empathy and thorough history taking were also noted as vital for building trust and rapport with young patients and their families.

*Resistance*

Resistance from families in relation to diagnosis and management was a significant concern. Physicians identified stigma associated with mental health, and resistance from parents as challenges that often hinder the implementation of best practices and the provision of optimal care.

*Commitment*

There was a strong call for continuous monitoring of child and adolescent psychiatry cases. Concerns were highlighted for inconsistent follow-up. These might reflect a lack of commitment from physicians or from families’ side, or some other factors that interfere with continuous care.

*Physician-Patient-Parent (P-P-P) Relationship*

The interaction between doctors, patients, and parents is essential for successful mental healthcare. Doctors discussed the intricacies of these interactions, emphasizing obstacles such as a perceived lack of privacy, anxiety, and family worries that prevent patients from sharing their concerns and receiving the assistance they require. Mutual respect, comprehension, and cooperation are necessary to achieve the best results for children and adolescents.

#### 3.2.3 Theme: clinic

This theme focuses on the physical and organizational environment of the clinic where primary mental health care is provided, highlighting factors that influence the delivery of care.

*Setting*

Physicians emphasized the importance of a conducive clinical setting that supports privacy and comfort for children and adolescents.

#### 3.2.4 Theme: system

This theme addresses the broader systemic factors that impact the practice of child and adolescent mental health care, including barriers, support, and interprofessional collaboration.

*Time*

Time constraints were a significant concern. Family physicians reported that limited consultation time often compromised the quality of care they could provide. They advocated for longer appointment slots and flexible scheduling for specialized care to better address the complex needs of children and adolescents with mental health issues.

*Resources*

The availability and adequacy of resources were frequently mentioned. Family physicians highlighted the need for sufficient diagnostic tools, therapeutic materials, and access to medications to deliver comprehensive and effective primary mental health care.

*Interprofessional Collaboration*

The importance of collaboration among healthcare professionals was emphasized for providing comprehensive care. Family physicians discussed the advantages and challenges of cooperating with psychiatrists, psychologists, social workers, pediatricians, and other specialists to offer coordinated and comprehensive care to young patients, particularly regarding the challenges of referring patients. They also highlighted the benefits of adopting a collaborative approach to care.

### 3.3 Patient data analysis

The dataset encompasses 382 valid entries from various family medicine clinics, detailing demographic, clinical, and referral information. In terms of gender, 59.9% of the patients were coded as male and 40.1% as female. Clinics with the highest patient volumes included Nad Al Hamar Clinic (24.5%) and Al Barsha Clinic (19.3%). Patient data analysis revealed a wide range of ICD-10 diagnoses of psychiatric conditions presenting to primary care (16). The most frequent clinical diagnoses codes included F84.0 (Autism Spectrum Disorder) at 40.1%, F41.9 (Anxiety Disorder, Unspecified) at 14.4%, F80.9 (Developmental disorder of speech and language) at 7.8%, F32.9 (Depressive episode, unspecified) at 4.2% and F90.2 (Attention-deficit hyperactivity disorder, combined type) at 4.2%.

In addition to these, family physicians have encountered a wide range of ICD-10 diagnoses of psychiatric conditions, including F90.0 Attention-deficit hyperactivity disorder, predominantly inattentive type (1%), F90.1 Attention-deficit hyperactivity disorder, predominantly hyperactive type (2.1%), and F90.8 Attention-deficit hyperactivity disorder, other type (0.3%). Language Disorders were as per the following: F80.1 Expressive language disorder (0.8%), F80.2 Mixed receptive-expressive language disorder (0.3%), and R47.9 Unspecified speech disturbances (0.3%).

The ICD-10 diagnoses for Anxiety Disorders were as per the following: F40.10 Social phobia, unspecified (1%), F41.0 Panic disorder [episodic paroxysmal anxiety] (2.9%), F41.1 Generalized anxiety disorder (0.5%), F41.8 Other specified anxiety disorders (0.5%), and F43.0 Acute stress reaction (0.3%). Mood Disorders as per the following: F32.89 Other specified depressive episodes (0.3%), F93.8 Other childhood emotional disorders (0.3%).

Obsessive-compulsive and related disorders were as per the following: F42.2 Mixed obsessional thoughts and acts (0.5%), F42.8 Other obsessive-compulsive disorder (0.5%), and F42.9 Obsessive-compulsive disorder, unspecified (1%). Psychotic Disorders were as follows: F20.0 Paranoid schizophrenia (0.3%), F20.9 Schizophrenia, unspecified (0.3%), F25.9 Schizoaffective disorder, unspecified (0.3%). Eating Disorders were as follows: F50.0 Anorexia nervosa (1%), F50.01 Anorexia nervosa, restricting type (0.3%), F50.2 Bulimia nervosa (0.3%), F50.81 Binge eating disorder (0.3%), F50.89 Other specified eating disorder (1%), F50.9 Eating disorder, unspecified (3.9%). In 38 cases (9.9%) the clinical diagnosis was missing.

Referral patterns show that 63.1% of the cases involved referrals, primarily to adolescent mental health services (29.0%) and general pediatrics (6.5%). A significant proportion was referred to local tertiary child and adolescent psychiatry care center. Subsequent visits with family medicine were high, with 72.8% of patients returning for follow-up.

The distribution of referrals by clinical diagnosis shows distinct patterns. Speech disorders (89%) and depression (81%) have the highest referral rates, followed by autism spectrum disorder (76%). Anxiety Disorder, Unspecified (F41.9), also shows a notable referral rate, indicating a strong association between this diagnosis and the need for further referrals. Other diagnoses, such as schizophrenia (F20.0) and ADHD, exhibit lower referral rates in comparison. Chi-square tests confirm a statistically significant relationship between the clinical diagnosis and the likelihood of a referral being made (*p*-value < 0.001) (Table 3). Follow-up visit rates, however, show a different trend, with ADHD (100%) and depression (94%) having the highest rates, while autism (54%) and speech disorders (60%) have the lowest rates.

**TABLE 3:** Correlation of most common diagnoses in patient data to referral and follow-up rates (*n* = 382).

Variable	Clinical diagnosis						Total	*p*-value for the Pearson’s Chi-square test*
	**F84.0 (autistic disorder)**	**F41.9 (unspecified anxiety disorder)**	**F 80.9 (developmental disorder of speech and language)**	**F90.2 (attention-deficit hyperactivity disorder, combined type)**	**F32.9 (depressive episode, unspecified)**	Other**		
**Referral made**								
Yes	117	34	26	8	13	43	241	<0.001
No	36	20	3	8	3	71	141382	
Total	153	54	29	16	16	114	241	
**Follow-up in PHCs**								
Yes	83	48	18	16	15	99	279	<0.001
No	70	7	12	0	1	14	104	
Total	153	55	30	0	16	113	382	

*A significance level of *p* ≥ 0.05 was established.

**Other ICD-10 codes: F20.0, F20.9, F25.9, F32.89, F40.10, F41.0, F41.1, F41.8, F42.2, F42.8, F42.9, F43.0, F50.00, F50.01, F50.2, F50.81, F50.89, F50.9, F80.1, F80.2, R47.9, F90.0, F90.1, F90.8, F93.8.

## 4 Discussion

Our study provides significant insights into the perceptions, barriers, and systemic challenges faced by primary care physicians (PCPs) in managing child and adolescent psychiatric conditions within the primary care setting in Dubai. The findings underscore the necessity for enhanced training, systemic reforms, and better resource allocation to improve the integration of mental health services in primary care. Patient data reflected knowledge gaps and referral patterns reported by family physicians, emphasizing the need for comprehensive training for family doctors.

### 4.1 Current situation in Dubai Health facilities

The present study offers a comprehensive analysis of the current state of family physicians’ involvement and preparedness in managing child and adolescent psychiatric care within Dubai Health. The survey response rate of 30.6%, although modest, is typical for survey-based studies in healthcare settings. The demographic breakdown, with a predominance of female respondents (82.5%), reflects the gender distribution commonly observed in family medicine globally (17).

The reliability of the survey was not assessed using Cronbach’s alpha due to the diverse structures of the questions, which included dichotomous and Likert-type items with varying response scales. This diversity rendered traditional measures of internal consistency inappropriate. As a result, we did not conduct any item-total analysis or exploratory factor analysis.

The survey findings underscore a considerable gap in formal training in child and adolescent psychiatry among family physicians, with only 33.3% receiving such training during their medical education and a mere 21.1% participating in CME activities. This deficiency is reflected in the self-reported preparedness levels, where a combined 54.4% of respondents felt not prepared or not very prepared to manage psychiatric care in young patients. These results align with previous literature suggesting a pervasive lack of psychiatric training among primary care physicians, which adversely impacts their confidence and effectiveness in managing these cases (18).

The frequency of encountering child and adolescent patients with psychiatric concerns varied widely among respondents, with 29.8% encountering such cases frequently (1–3 times per month) and another 29.8% occasionally (1–3 times per year). Notably, 86% of the physicians conducted a primary assessment and referred to specialized care if needed, reflecting a tendency to defer to specialists due to perceived inadequacies in their training.

### 4.2 Analysis of patient data

The analysis of patient data from family medicine clinics in Dubai Health reveals several key findings about the demographic, clinical, and referral patterns in child and adolescent psychiatric care and mirrors self-reported data from the survey. The patient demographic data indicate a higher proportion of male patients (59.9%) compared to female patients (40.1%). This gender disparity might reflect underlying differences in the prevalence or recognition of psychiatric conditions among boys and girls. It is consistent with existing literature, particularly concerning conditions like Autism Spectrum Disorder (ASD) and Attention-Deficit/Hyperactivity Disorder (ADHD), which are more commonly diagnosed in males (19, 20).

The most frequent diagnosis among the patients was Autism Spectrum Disorder (F84.0), accounting for 43.1% of the cases. This high prevalence underscores the significant demand for autism-related services and the need for specialized care pathways in family medicine clinics. Anxiety Disorder, Unspecified (F41.9) and Developmental Disorder of Speech and Language (F80.9) were also prevalent, indicating a broad spectrum of psychiatric concerns being managed at the primary care level. The relatively lower prevalence of Depressive Episode, Unspecified (F32.9) and ADHD, Combined Type (F90.2) suggests potential underdiagnosis or referral patterns that prioritize certain conditions over others. These diagnosis frequencies correspond with the self-reported topics for training outlined by family physicians in the survey: attention deficit hyperactivity disorder, anxiety disorders, autism spectrum disorder, depressive disorders, and eating disorders.

The high rate of referrals (63.1%), for diagnoses like Autism Spectrum Disorder (F84.0) and Anxiety Disorder, Unspecified (F41.9), indicates a reliance on specialized care. This pattern suggests that while family physicians are often the first point of contact, they predominantly act as gatekeepers rather than primary managers of psychiatric conditions in children and adolescents. The statistically significant relationship between clinical diagnoses and referral likelihood underscores the critical need for enhanced training to equip family physicians to handle more cases independently.

The frequent follow-up visits (72.8%) highlight the ongoing role of family physicians in managing psychiatric conditions, even after referral. This continuity of care is essential for ensuring comprehensive management and support for these patients and their families.

### 4.3 Qualitative analysis of open-ended questions

The qualitative analysis of open-ended questions enabled a more detailed identification of existing challenges:

*Time Constraints*

Time constraints remain a major barrier, as PCPs report insufficient time to address psychiatric issues comprehensively. This is consistent with studies indicating that high patient volumes and numerous responsibilities limit the ability to provide adequate mental health care (4, 6, 21). Qualitative data revealed that PCPs often feel rushed during consultations, making it difficult to perform thorough psychiatric evaluations and establish a therapeutic relationship with young patients. They reported that the complexity of psychiatric issues requires more time than the standard appointment slot allows, leading to feelings of frustration and inadequacy in providing comprehensive care.

*Lack of Sophistication in Psychiatric Knowledge*

Many PCPs feel that their training does not equip them with the sophisticated knowledge needed to diagnose and treat psychiatric disorders effectively. This gap necessitates specialized training and support, as highlighted by collaborative and integrated care models (22, 23). Our data showed that 66.7% of respondents did not receive any training in child and adolescent psychiatry during their formal education, and 78.9% did not receive CME training, indicating significant gaps in education and ongoing professional development.

*Resistance to Mental Health Services*

Resistance to mental health services in general and within primary care is evident due to stigma, lack of confidence, and organizational inertia. Additionally, some parents may resist accepting that their children have a psychiatric disorder or disagree with the recommended treatment plans, which adds another layer of complexity to care provision. This resistance must be addressed to facilitate the important role of PCPs in collaborative care models in the context of scarcity in child and adolescent psychiatrists (24).

*Systemic and Structural Barriers*

Inadequate healthcare infrastructure and insufficient reimbursement for mental health services pose significant challenges (25). These systemic issues limit PCPs’ ability to provide comprehensive psychiatric care. Only 24.6% of our respondents participated in collaborative sessions between primary and secondary care physicians, highlighting structural barriers to effective interdisciplinary collaboration.

*Communication and Coordination*

Effective communication and coordination between PCPs, mental health specialists, and other stakeholders are crucial. Improving these aspects can enhance collaborative care models’ effectiveness (26). The qualitative data emphasized the importance of clear, empathetic communication, especially in conveying complex psychiatric information to patients and families.

*Resource Limitations*

Limited access to specialized mental health professionals and diagnostic tools significantly hampers PCPs’ ability to manage psychiatric conditions. Ensuring primary care settings are well-equipped is vital. Our study showed that 86.0% of PCPs conducted a primary assessment and referred to specialized care if needed, but only a minority managed cases themselves, indicating a reliance on external resources that are often scarce.

*Improving Capacity and Resources*

Enhancing PCPs’ capacity to manage pediatric psychiatric conditions involves comprehensive training and continuous education. Implementing collaborative care models that provide ongoing specialist support is essential. Policy reforms are necessary to improve systemic infrastructure and resource allocation (27). The high interest (89.5%) in collaborative care educational initiatives among our respondents suggests a strong demand for these resources.

### 4.4 Primary care physicians’ perceptions toward collaborative care

Primary care physicians generally recognize the benefits of collaborative care models, which include improved patient outcomes and more comprehensive care. However, many feel unprepared and lack confidence in managing psychiatric conditions. This aligns with findings from Green et al. (22) and Hoffmann et al. (28), who emphasize the importance of integrated care and continuous specialist support in primary care settings. In our study, only 5.3% of PCPs felt very prepared to manage child and adolescent psychiatric care, underscoring the need for enhanced training and support.

### 4.5 Strengths and limitations

This study’s strengths include its mixed-methods design, providing a holistic understanding of PCPs’ challenges. However, potential response bias and the study’s focus on a specific geographical area may limit generalizability.

The representativeness of our sample is another important consideration when interpreting the findings of this study. While our sample of PCPs in Dubai provides valuable insights, it may not fully capture the diversity of the broader population of PCPs working in this region. Factors such as the size of the sample, selection criteria, and the specific healthcare settings in which participants were employed could limit the generalizability of our results. As Dubai’s healthcare landscape is diverse, with professionals from various cultural, educational, and clinical backgrounds, a larger and more diverse sample would offer a more comprehensive understanding of the challenges and needs faced by PCPs across different settings. Consequently, caution should be exercised when applying these findings to all PCPs in Dubai. Future research should aim to include a broader range of participants from various healthcare institutions to enhance the generalizability of the results.

One limitation of this study is the absence of formal validation procedures for the survey instrument beyond content validation. Although the survey’s content validity was ensured through expert input, other validation methods, such as construct validity or confirmatory factor analysis, were not conducted. The lack of additional validation techniques may limit the confidence in the generalizability of the survey results. Future studies should incorporate more comprehensive validation methods, such as pilot testing or applying construct validation techniques, to enhance the robustness and applicability of the survey across various contexts and populations.

### 4.4 Implications for practice and policy

The findings have significant implications for both practice and policy. Developing evidence-based training programs and implementing collaborative care models can improve psychiatric condition management in children and adolescents within primary care. Systemic reforms to address structural barriers and ensure equitable access to mental health resources are essential. The findings suggest several key areas for improvement. There is an urgent need for enhanced training programs in child and adolescent psychiatry within medical education and through CME activities. Developing structured, collaborative care models that integrate family physicians with psychiatric specialists could improve care coordination and outcomes. Additionally, addressing systemic barriers, such as time constraints and resource limitations, is crucial.

Efforts to reduce stigma and enhance parental engagement in psychiatric care are also vital. Providing family physicians with tools and training to effectively communicate and manage these dynamics can improve the overall treatment experience for patients and their families.

### 4.5 Future research directions

Future research should address gaps in the current understanding of PCPs’ challenges in managing pediatric psychiatric conditions. Longitudinal studies and broader demographic coverage are needed to enhance the robustness of findings and support evidence-based interventions. Additionally, it is crucial to evaluate family satisfaction with collaborative care and to compare mental health outcomes between patients managed with collaborative care and those managed with standard referral-based care.

## 5 Conclusion

Our study highlights the critical need for enhanced training and support for PCPs in managing child and adolescent psychiatric conditions. By addressing the identified barriers through collaborative care models, continuous education, and systemic reforms, we can improve the quality of mental health care provided in primary care settings and ensure better outcomes for children and adolescents with psychiatric conditions.

## Data Availability

The original contributions presented in this study are included in this article/Supplementary material, further inquiries can be directed to the corresponding author.
