# Supplementary material for: Empowering primary care physicians in child and adolescent psychiatry: a needs assessment on collaborative care in Dubai
Source: Front Med (Lausanne). 2024 Nov 11;11:1456212. doi: 10.3389/fmed.2024.1456212 (PMC11586184; doi:10.3389/fmed.2024.1456212)
Supplement: Supplementary file 1 [file Data_Sheet_1.pdf]

Supplementary Material

S1. Survey structure. **-indicates questions that are required to answer if a mentioned condition is satisfied.*

N	Question	Variables
1.	Please, define your gender:	Male<br>Female
2.	Please, define your age:	Under 30<br>30-39<br>40-49<br>50-59<br>60 or older
3.	How many years have you been practicing as a family physician?	Number
4.	Do you primarily work with pediatric patients, adult patients, or a mix of both?	Pediatric patients mostly<br>Adult patients mostly<br>Mix of pediatric and adult patients
5.	Which clinic do you work at?	Drop-down menu (Al Badaa, Al Barsha, Al Khawaneej, Al Lusaily, Al Mamzar, Al Mankhool, Al Mizhar, Al Qusais, Al Towar, Nad Al Hamar, Nad Al Sheba, Tele-Medicine, Umm Suqeim, Za'abeel)
6.	Have you received specific training or education in child and adolescent psychiatry during your medical school/residency/fellowship?	Yes<br>No
7.	If yes, please, briefly describe the nature and extent of this training*:	Open-ended
8.	Have you received specific training or education in child and adolescent psychiatry as part of continuing medical education (CME)?	Yes<br>No
9.	If yes, please, briefly describe the nature and extent of this training*:	Open-ended
10.	Have you ever been involved in collaborative sessions between primary and secondary care physicians?	Yes<br>No<br>Do not remember

11.	If yes, please, briefly describe the topics covered during collaborative sessions*:	Open-ended
12.	How often do you encounter child and adolescent patients presenting with psychiatric concerns in your practice?	Never<br>Occasionally (1-3 times per year)<br>Sometimes (around once a month)<br>Frequently (1-3 times per month)<br>Very frequently (weekly)
13.	When you encounter a child or adolescent patient with significant psychiatric needs, what is your typical course of action?	Manage the case myself<br>Conduct primary assessment and refer to specialized care if needed<br>Refer to specialized care immediately<br>Other (open-ended)
14.	What percent of cases do you refer to specialized care?*	0-15%<br>15-30%<br>30-50%<br>Over 50%
15.	Where do you most frequently refer a child or adolescent patient with significant psychiatric needs?	To a child and adolescent psychiatrist<br>Refer to a pediatrician<br>Refer to a mental health counselor or therapist<br>Other (open-ended)
16.	Do you feel adequately prepared to manage child and adolescent psychiatric care in your practice?	Very prepared<br>Somewhat prepared<br>Not very prepared<br>Not prepared at all
17.	What specific challenges or barriers do you face when managing child and adolescent psychiatric care in your practice?	Open-ended
18.	What topics or areas related to child and adolescent psychiatry would you be interested in receiving additional training or education on?	Multiple choice:<br>Attention-deficit/ Hyperactivity Disorder (ADHD)<br>Autism Spectrum Disorder<br>Anxiety Disorders<br>Depressive Disorders<br>Suicide Risk Assessment<br>Obsessive-compulsive Disorder<br>Tic Disorders<br>Bipolar Disorders<br>Psychotic Disorders<br>Eating Disorders<br>Other (open-ended)
19.	Would you be interested in collaborative care educational initiatives or interdisciplinary teams for managing child and	Yes<br>No<br>Maybe

	adolescent psychiatric care in your practice?	
20.	Please provide any additional comments or suggestions related to improving your capability to manage child and adolescent psychiatric care as a family physician:	Open-ended

S2. Patient data extraction tool with codes explanation

**MIS code**	**Description**
MASKED_PATIENT	Patient ID
MASKED_EPISODE	Episode
LOC_NAME	Clinic
DEPARTMENT_NAME	Department
SPECIALTY	Specialty
DT	Date
AGE	Patient age
GENDER_CODED	Patient gender
PROV_ID	Provider ID
PROV_NAME	Provider name
PROV_TYPE	Provide type
CLINICAL_DX	Clinical diagnosis
HAR_DX	Hospital admission request diagnosis
REFERRAL_MADE_CODED	Referral made (yes/no)
REFERRAL_ORDER_DESCRIPTION	Referral facility
SUBSEQUENT VISIT WITH FAMILY MEDICINE_CODED	Follow-up visit at PHC (yes/no)
